# A new distribution record, first host plant record and DNA barcoding of the Neotropical micromoth *Astrotischeriakarsholti* Puplesis & Diškus (Lepidoptera, Tischeriidae)

**DOI:** 10.3897/BDJ.11.e115397

**Published:** 2023-12-19

**Authors:** Héctor A. Vargas

**Affiliations:** 1 Universidad de Tarapacá, Facultad de Ciencias Agronómicas, Departamento de Recursos Ambientales, Arica, Chile Universidad de Tarapacá, Facultad de Ciencias Agronómicas, Departamento de Recursos Ambientales Arica Chile

**Keywords:** Asteraceae, Atacama Desert, DNA barcodes, host plant, leaf-mining larvae

## Abstract

**Background:**

*Astrotischeria* Puplesis & Diškus, 2003 (Lepidoptera, Tischeriidae) is a New World genus of micromoths whose larvae are leaf miners associated mainly with plants of the family Asteraceae. The original description of the type species *Astrotischeriakarsholti* Puplesis & Diškus, 2003 was based on adults from central Peru. No additional distribution records, host plants or DNA barcodes have been documented for this species.

**New information:**

*Astrotischeriakarsholti* is reported for the first time from Chile, based on adults obtained from leaf mines of *Ambrosiacumanensis* Kunth (Asteraceae) collected in the transverse valleys of the Atacama Desert. This discovery expands the distribution range of this micromoth nearly 900 km to the southeast and represents its first host plant record. Divergence between DNA barcodes of *A.karsholti* and the nearest congeneric was 6% (K2P). A Maximum Likelihood analysis, based on DNA barcodes, raises questions about the monophyly of *Astrotischeria*.

## Introduction

The widespread micromoth family Tischeriidae (Lepidoptera) currently includes 186 species described worldwide, grouped in 11 genera (Puplesis and Diškus 2003, Stonis et al. 2023). Plants belonging to 22 families have been recorded as hosts for their leaf-mining larvae, although each species has a narrow host range (Puplesis and Diškus 2003, Stonis et al. 2018, Xu et al. 2021, Stonis et al. 2023) . The New World genus *Astrotischeria* Puplesis & Diškus, 2003 can be differentiated from other genera of Tischeriidae by morphological characteristics of the genitalia (Puplesis and Diškus 2003), amongst which the uncus with four lobes and the valva with at least one elaborated dorsal lobe in the male are the most striking (Stonis et al. 2023). Thirty Nearctic and Neotropical species were originally included in this genus, many of which were previously known as members of *Tischeria* Zeller, 1839 (Puplesis and Diškus 2003). Subsequent studies raised the number of described species to 45 and revealed many others that remain undescribed (Landry and Roque-Albelo 2004, Stonis et al. 2018, Stonis et al. 2019a, Stonis et al. 2019b, Stonis et al. 2020a, Stonis et al. 2020b, Stonis et al. 2020c, Stonis et al. 2023). The main host plant family of *Astrotischeria* is Asteraceae, while a few species are associated with members of Malvaceae (Puplesis and Diškus 2003, Stonis et al. 2023).

The only representative of *Astrotischeria* recorded in Chile is the endemic *Astrotischeriachilei* Puplesis & Diškus, 2003, whose original description was based on two male adults (holotype and paratype) collected in the southern locality of Los Alpes (Angol) in the Malleco Province (Puplesis and Diškus 2003). Additional adults of both sexes were subsequently reared from leaf mines of the Chilean endemic shrub *Podanthusovatifolius* Lag. (Asteraceae) collected in Río Clarillo National Park in central Chile, a discovery that revealed the only host plant recorded so far, expanded the previously documented distribution about 500 km to the north and allowed the first description of the female (Stonis et al. 2016).

*Astrotischeriakarsholti* Puplesis & Diškus, 2003, type species of *Astrotischeria*, was originally described from central Peru, based on the male holotype from Huangascar and male and female paratypes from the same locality and Matucana in the Lima Department and Huancayo in the Junin Department (Puplesis and Diškus 2003). No additional distribution records or field observations of this species have been documented after its original description. However, recent fieldwork in northern Chile resulted in the discovery of *A.karsholti* and its host plant in this country. The aim of this contribution is to provide these records and to assess the relationship of *A.karsholti* to congeneric species using mitochondrial DNA sequences.

## Materials and methods

The micromoths examined were obtained from leaf mines of *Ambrosiacumanensis* Kunth (Asteraceae) collected in the Azapa Valley (18°31’19’’ S, 70°10’42’’ W), Arica Province of northern Chile, at about 260 m elevation. The abdomen of each specimen was removed and boiled in 10% potassium hydroxide (KOH) for a few minutes for dissection of the genitalia, which were stained with Eosine Y and mounted on slides with Euparal. Voucher specimens and their respective genitalia slides are deposited in the “Colección Entomológica de la Universidad de Tarapacá” (IDEA), Arica, Chile. The distribution map was generated using SimpleMappr (Shorthouse 2010).

Genomic DNA was extracted from one pupa following the procedures described in Huanca-Mamani et al. (2015). DNA purification, PCR amplification and sequencing of the barcode fragment (Hebert et al. 2003) were performed in Macrogen Inc. (Seoul, South Korea) with the primers LCO1490 and HCO2198 (Folmer et al. 1994). The PCR programme was 5 min at 94°C, 35 cycles of 30 s at 94°C, 30 s at 47°C, 1 min at 72°C and a final elongation step of 10 min at 72°C. The sequence obtained was deposited in BOLD (Ratnasingham and Hebert 2007) under Process ID NCMIC004-23.

Full length DNA barcodes of *Astrotischeria* provided by Stonis et al. (2023) were downloaded from GenBank (Benson et al. 2012) or BOLD to assess the relationships of *A.karsholti*. The sampling also included sequences of *Gnathitischeria* Diškus, 2023 and *Paratischeria* Diškus & Stonis, 2017, due to their closeness to *Astrotischeria* (Stonis et al. 2023), *Tischeria*, since this genus previously harboured some species currently ascribed to *Astrotischeria* (Puplesis and Diškus 2003), *Coptotriche* Walsingham, 1890, due to its early divergence within the family (Stonis et al. 2023) and *Azaleodes* Turner, 1923 (Palaephatidae) as a representative of the sister group of Tischeriidae (Regier et al. 2015, Bazinet et al. 2016). The final dataset included 26 DNA barcodes of 657 base pairs (Suppl. material 1). The software MEGA 11 (Tamura et al. 2021) was used to perform sequence alignment with the ClustalW method, to translate nucleotides into amino acids and to assess sequence divergence with the Kimura 2-Parameter (K2P) method. Substitution saturation was estimated with the Xia test (Xia et al. 2003) in DAMBE 7 (Xia 2018). No evidence of stop codons was detected and the Xia test revealed an index of substitution saturation less than the critical value in the alignment (ISS < ISS.C; p < 0.001). A Maximum Likelihood (ML) tree was inferred using IQTREE 1.6.12 (Nguyen et al. 2015) in the web interface W-IQ-TREE (Trifinopoulos et al. 2016) with the data partitioned to codon position. TN+F+I, F81+F+I and HKY+F+G4 were selected as the best-fit models for 1^st^, 2^nd^ and 3^rd^ partitions, respectively, in ModelFinder (Kalyaanamoorthy et al. 2017). Branch support was assessed with 1,000 replications of the Shimodaira-Hasegawa-like approximate likelihood ratio test (SH-aLRT) (Guindon et al. 2010) and ultrafast bootstrap (UFBoot) (Hoang et al. 2017). The unrooted tree was visualised in FigTree (Rambaut 2014) to root on *Azaleodes*.

## Taxon treatments

### Astrotischeria karsholti

Puplesis & Diškus, 2003

3A537BAE-D582-569A-A57D-25301B1D36E4

#### Materials

- **Type status:** Other material. **Occurrence:** individualCount: 2; sex: male; occurrenceID: 4FCB30DA-EF41-5959-B20D-21C560A30886; **Taxon:** scientificName: *Astrotischeriakarsholti* Puplesis & Diškus, 2003; higherClassification: Insecta; Lepidoptera; Tischeriidae; genus: Astrotischeria; specificEpithet: karsholti; scientificNameAuthorship: Puplesis & Diškus, 2003; **Location:** continent: South America; country: Chile; stateProvince: Arica; locality: Azapa Valley; decimalLatitude: -18.52; decimalLongitude: -70.18; **Identification:** identifiedBy: Héctor A. Vargas; identificationRemarks: Genitalia slides HAV1680, HAV1681; **Event:** samplingProtocol: Male adults emerged October 2022, reared from leaf mines on Ambrosiacumanensis collected September 2022; **Record Level:** type: PhysicalObject; language: en; institutionID: "Colección Entomológica de la Universidad de Tarapacá" (IDEA)
- **Type status:** Other material. **Occurrence:** individualCount: 2; sex: male; occurrenceID: 98E275BB-728F-59D6-872A-79CFAFA3B21E; **Taxon:** scientificName: *Astrotischeriakarsholti* Puplesis & Diškus, 2003; higherClassification: Insecta; Lepidoptera; Tischeriidae; genus: Astrotischeria; specificEpithet: karsholti; scientificNameAuthorship: Puplesis & Diškus, 2003; **Location:** continent: South America; country: Chile; stateProvince: Arica; locality: Azapa Valley; decimalLatitude: -18.52; decimalLongitude: -70.18; **Identification:** identifiedBy: Héctor A. Vargas; identificationRemarks: Genitalia slides HAV1088, HAV1405; **Event:** samplingProtocol: Male adults emerged November 2017, reared from leaf mines on Ambrosiacumanensis collected October 2017; **Record Level:** type: PhysicalObject; language: en; institutionID: "Colección Entomológica de la Universidad de Tarapacá" (IDEA)
- **Type status:** Other material. **Occurrence:** individualCount: 1; sex: male; occurrenceID: 831F005C-C567-5180-914C-B8D68CA49522; **Taxon:** scientificName: *Astrotischeriakarsholti* Puplesis & Diškus, 2003; higherClassification: Insecta; Lepidoptera; Tischeriidae; genus: Astrotischeria; specificEpithet: karsholti; scientificNameAuthorship: Puplesis & Diškus, 2003; **Location:** continent: South America; country: Chile; stateProvince: Arica; locality: Azapa Valley; decimalLatitude: -18.52; decimalLongitude: -70.18; **Identification:** identifiedBy: Héctor A. Vargas; identificationRemarks: Genitalia slide HAV119; **Event:** samplingProtocol: Male adult emerged April 2018, reared from leaf mines on Ambrosiacumanensis collected March 2018; **Record Level:** type: PhysicalObject; language: en; institutionID: "Colección Entomológica de la Universidad de Tarapacá" (IDEA)

#### Taxonomic identification

**Figure 1.**
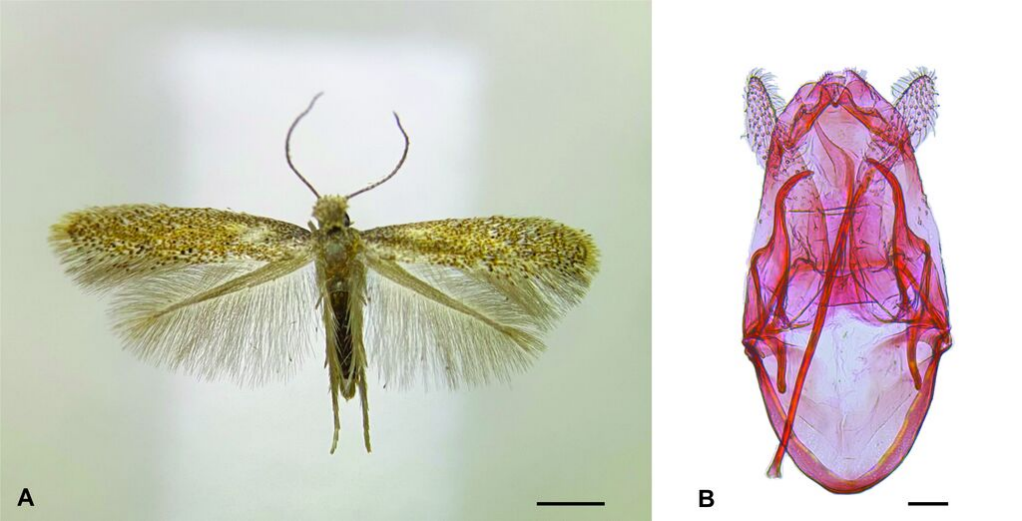
Male adult of *Astrotischeriakarsholti* Puplesis & Diškus, 2003 reared from a leaf mine on *Ambrosiacumanensis* Kunth (Asteraceae) collected in northern Chile. **A** Habitus, dorsal view; **B** Genitalia, ventral view. Scale bars 1 and 0.01 mm, respectively.

Five male adults emerged from the mined leaves of *A.cumanensis* collected in the Azapa Valley, all of which were identified as *A.karsholti* (Fig. 1), based on comparisons with descriptions and figures in Puplesis and Diškus (2003) and Stonis et al. (2018).

#### Distribution

**Figure 2.**
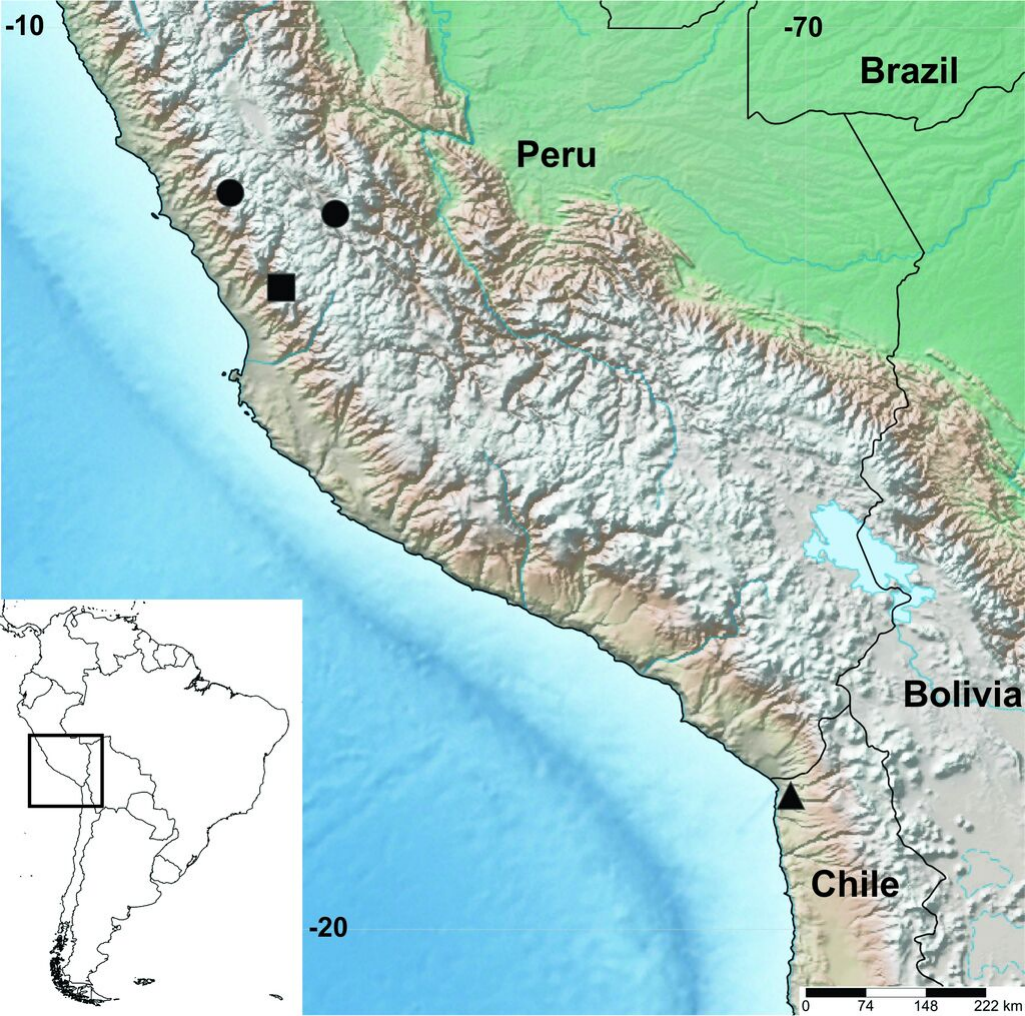
Geographic distribution of *Astrotischeriakarsholti* Puplesis & Diškus, 2003 in South America. Previous records from central Peru indicated by square (type locality) and circles, new record from northern Chile indicated by triangle.

The discovery of *A.karsholti* in the Azapa Valley represents the first record of this micromoth in Chile, expanding the previously documented distribution range nearly 900 km to the southeast (Fig. 2).

#### Host plant

**Figure 3.**
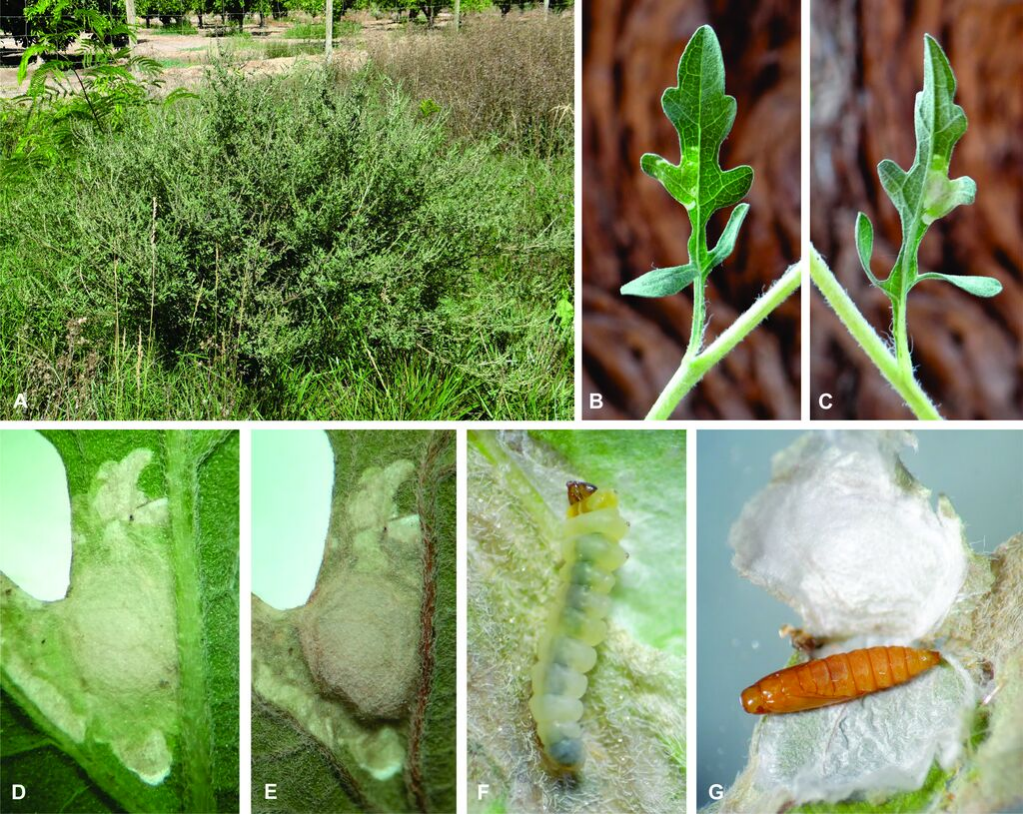
Natural history of *Astrotischeriakarsholti* Puplesis & Diškus, 2003 in the Azapa Valley, Arica Province of northern Chile. **A** The host plant *Ambrosiacumanensis* Kunth (Asteraceae) in the neighbourhood of a citrus orchard; **B, C** Leaf mine on *A.cumanensis* previous to nidus formation, adaxial and abaxial views, respectively; **D, E** Leaf mine at the beginning and at the end of nidus formation, respectively, abaxial view; **F** Last instar larva removed from mine of B and C; **G** Nidus of D and E opened to show the pupa.

*Ambrosiacumanensis* is the first host plant recorded for *A.karsholti*. Leaf mines of *A.karsholti* were searched for on other members of Asteraceae growing in the study area, but no additional hosts were found for this micromoth. The egg is deposited on the abaxial surface of the leaf and the larva penetrates the leaf through this side. New mines are visible only from the abaxial surface of the leaf, while completely developed mines are partially translucent and, thus, detectable from the two leaf sides, suggesting that the larva eats a great part of the internal tissues of the leaf. The last instar constructs a well-delimited circular cell (nidus) inside the mine for pupation. Adult emergence occurs through a slit on the margin of the nidus (Fig. 3).

#### DNA barcoding

**Figure 4.**
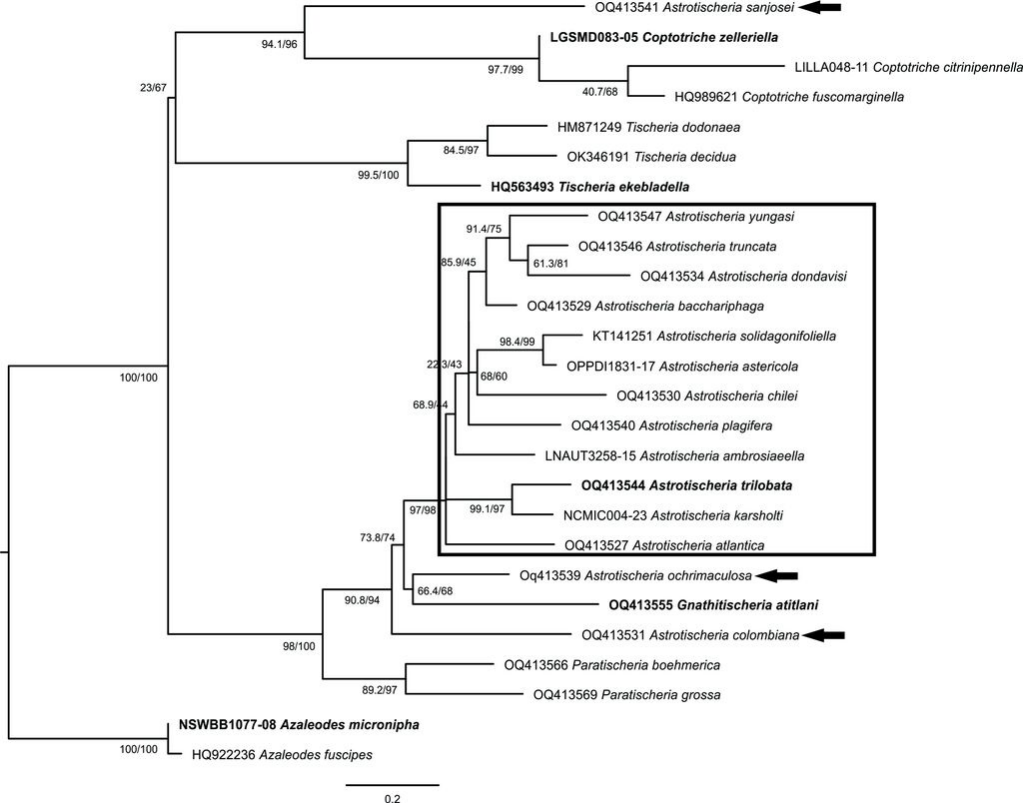
Maximum Likelihood tree of *Astrotischeriakarsholti* Puplesis & Diškus, 2003 and other members of Tischeriidae, based on mitochondrial DNA sequences. Rectangle indicates a monophyletic group of *Astrotischeria* Puplesis & Diškus, 2003; species of this genus clustered outside this clade are indicated by black arrows; type species are in bold. Numbers indicate SH-aLRT/UFBoot values (1000 replicates).

Genetic divergence of *A.karsholti* with other members of *Astrotischeria* ranged between 6 and 18.4% (K2P), with *Astrotischeriatrilobata* Diškus & Stonis, 2018 and *Astrotischeriasanjosei* Stonis & Diškus, 2019, respectively, while it was 9.9% with *A.chilei*, the only Chilean congeneric (Suppl. material 2). The monophyly of *Coptotriche*, *Paratischeria* and *Tischeria* was strongly supported in the ML analysis (Fig. 4). In contrast, only 12 of the 15 analysed species of *Astrotischeria* formed a monophyletic group. The relationships in this group were poorly resolved, with the exception of *A.karsholti* + *A.trilobata* and *Astrotischeriasolidagonifoliella* (Clemens, 1859) + *Astrotischeriaastericola* (Braun, 1972).

## Discussion

The discovery of *A.karsholti* in the Azapa Valley increases to two the members of *Astrotischeria* recorded in Chile. As already indicated by Puplesis and Diškus (2003) and Stonis et al. (2016), this micromoth is accurately differentiated from the only Chilean congeneric *A.chilei* based on genitalia morphology. These two species also use different host plants and show deep DNA barcode divergence. Although the new distribution record of *A.karsholti* expands its range from central Peru to northern Chile, the two species remain clearly allopatric, because *A.chilei* is restricted to south-central Chile (Stonis et al. 2016).

The association of *A.karsholti* with Asteraceae reported here fits the most widespread host plant family previously documented for members of *Astrotischeria* (Puplesis and Diškus 2003, Stonis et al. 2023). The Nearctic *Astrotischeriaambrosiaeella* (Chambers, 1875) and *Astrotischeriaheliopsisella* (Chambers, 1875) were the only species of the genus previously known to be associated with *Ambrosia* (Puplesis and Diškus 2003, Stonis et al. 2020c). As *A.cumanensis* is widely distributed in Central and South America (Moreira-Muñoz et al. 2016, Luebert and Garcia 2020), further surveys for leaf mines in different environments in this extensive area would be extremely helpful to improve the understanding of the geographic distribution of *A.karsholti*.

Although the ML analysis was based on a single mitochondrial marker, similar procedures are generally useful for generic assignments of species of Lepidoptera (Moreira et al. 2012, Metz et al. 2019, Corley et al. 2020, San Blas et al. 2021, Wanke et al. 2022). In the present study, the lack of monophyly of *Astrotischeria* was an unexpected result. However, as already highlighted in their respective original descriptions, the three species grouped outside the *Astrotischeria* clade have remarkable morphological characteristics (Stonis et al. 2019a, Stonis et al. 2019b). Whether these peculiarities support different generic designations deserves further attention. The mostly poorly-resolved relationships of the species of the *Astrotischeria* clade could be due to the reduced taxon sampling that included only 15 of the 45 currently described species. Furthermore, Stonis et al. (2023) indicated that many additional undescribed species of this genus have been discovered. Although the clustering of *A.karsholti* with *A.trilobata* was strongly supported in the ML analysis, the 6% divergence suggests that these are not sister species. Further molecular phylogenetic studies, based on wider taxon sampling and additional molecular markers, would be extremely useful to improve the understanding of the delimitation of *Astrotischeria* and the relationships between its species.

## Supplementary Material

XML Treatment for Astrotischeria karsholti

DE4D0501-458E-5E1C-AE4B-58B3FD15FFC5 10.3897/BDJ.11.e115397.suppl1 10424630 Supplementary material 1 Alignment of DNA barcodes used in the ML analysis Data type DNA barcodes Brief description DNA barcodes of *Astrotrischeriakarsholti* and other Tischeriidae. File: oo_928882.fas https://binary.pensoft.net/file/928882 Héctor A. Vargas

91ABBFC6-7A04-5F4E-895D-345CAB58E702 10.3897/BDJ.11.e115397.suppl2 Supplementary material 2 Genetic distances of Astrotischeriakarsholti Data type Genetic distances (K2P) Brief description Genetic distances (K2P) of *Astrotischeriakarsholti*. File: oo_928883.pdf https://binary.pensoft.net/file/928883 Héctor A. Vargas
