# Supplementary material for: A new distribution record, first host plant record and DNA barcoding of the Neotropical micromoth Astrotischeriakarsholti Puplesis & Diškus (Lepidoptera, Tischeriidae)
Source: Biodivers Data J. 2023 Dec 19;11:e115397. doi: 10.3897/BDJ.11.e115397 (PMC10751789; doi:10.3897/BDJ.11.e115397)
Supplement: Supplementary material 2 — Genetic distances of Astrotischeriakarsholti [file bdj-11-e115397-s002.pdf]

	1	2	3	4	5	6	7	8	9	10	11	12	13	14
1 Astrotischeria_karsholti														
2 Astrotischeria_ambrosiaeella	**0,091**													
3 Astrotischeria_astericola	**0,094**	0,081												
4 Astrotischeria_atlantica	**0,103**	0,100	0,102											
5 Astrotischeria_bacchariphaga	**0,089**	0,090	0,077	0,089										
6 Astrotischeria_chilei	**0,099**	0,093	0,084	0,105	0,080									
7 Astrotischeria_colombiana	**0,133**	0,124	0,126	0,141	0,120	0,115								
8 Astrotischeria_dondavisi	**0,099**	0,098	0,094	0,101	0,089	0,097	0,131							
9 Astrotischeria_ochrimaculosa	**0,110**	0,096	0,115	0,121	0,108	0,117	0,121	0,107						
10 Astrotischeria_plagifera	**0,080**	0,079	0,086	0,116	0,086	0,097	0,131	0,100	0,110					
11 Astrotischeria_sanjosei	**0,184**	0,200	0,178	0,192	0,184	0,184	0,172	0,186	0,185	0,186				
12 Astrotischeria_solidagonifoliella	**0,101**	0,096	0,039	0,103	0,082	0,085	0,120	0,098	0,123	0,094	0,178			
13 Astrotischeria_trilobata	**0,060**	0,110	0,092	0,106	0,098	0,112	0,126	0,117	0,108	0,108	0,184	0,101		
14 Astrotischeria_truncata	**0,092**	0,089	0,086	0,081	0,064	0,079	0,120	0,072	0,108	0,089	0,182	0,086	0,103	
15 Astrotischeria_yungasi	**0,108**	0,103	0,094	0,112	0,086	0,098	0,137	0,091	0,121	0,098	0,198	0,102	0,130	0,079
16 Gnathitischeria_atitlani	0,139	0,140	0,128	0,152	0,131	0,144	0,143	0,146	0,126	0,133	0,196	0,137	0,120	0,135
17 Paratischeria_boehmerica	0,133	0,126	0,124	0,142	0,133	0,110	0,139	0,150	0,144	0,135	0,184	0,137	0,122	0,138
18 Paratischeria_grossa	0,159	0,145	0,164	0,167	0,154	0,146	0,154	0,157	0,141	0,152	0,208	0,171	0,148	0,161
19 Tischeria_decidua	0,166	0,155	0,146	0,157	0,157	0,148	0,151	0,151	0,161	0,159	0,210	0,155	0,159	0,148
20 Tischeria_dodonaea	0,172	0,163	0,148	0,168	0,159	0,159	0,153	0,164	0,159	0,170	0,220	0,159	0,155	0,148
21 Tischeria_ekebladella	0,170	0,172	0,170	0,174	0,155	0,166	0,170	0,171	0,169	0,182	0,184	0,178	0,163	0,155
22 Coptotriche_citrinipennella	0,166	0,190	0,170	0,166	0,170	0,170	0,159	0,168	0,161	0,178	0,180	0,174	0,168	0,176
23 Coptotriche_fuscomarginella	0,174	0,193	0,166	0,182	0,170	0,164	0,166	0,182	0,178	0,176	0,176	0,164	0,161	0,170
24 Coptotriche_zelleriella	0,174	0,180	0,161	0,172	0,161	0,166	0,168	0,188	0,188	0,166	0,172	0,170	0,166	0,164
25 Azaleodes_fuscipes	0,174	0,178	0,168	0,184	0,184	0,194	0,190	0,192	0,190	0,188	0,198	0,167	0,168	0,176
26 Azaleodes_micronipha	0,184	0,182	0,176	0,174	0,184	0,204	0,194	0,194	0,194	0,192	0,204	0,176	0,178	0,182

Genetic distances between Astrotischeria karsholti and other members of Tischeriidae and Palaephatic
The analysis used 657 base pairs of DNA barcodes under the Kimura 2-parameter model.
Genetic distances between representatives of Astrotischeria in yellow.
Genetic distances between A. karsholti and congeneric species in bold.

15	16	17	18	19	20	21	22	23	24	25
0,167										
0,155	0,140									
0,163	0,165	0,096								
0,159	0,161	0,155	0,159							
0,163	0,168	0,157	0,163	0,059						
0,186	0,174	0,167	0,178	0,085	0,089					
0,180	0,200	0,168	0,213	0,168	0,159	0,172				
0,182	0,195	0,161	0,201	0,178	0,164	0,174	0,080			
0,186	0,208	0,165	0,180	0,174	0,170	0,168	0,105	0,064		
0,190	0,196	0,165	0,174	0,178	0,182	0,172	0,174	0,168	0,182	
0,196	0,204	0,168	0,184	0,176	0,182	0,174	0,172	0,170	0,178	0,022

dae.
